# Sources of Error in Mammalian Genetic Screens

**DOI:** 10.1534/g3.116.030973

**Published:** 2016-07-06

**Authors:** Laura Magill Sack, Teresa Davoli, Qikai Xu, Mamie Z. Li, Stephen J. Elledge

**Affiliations:** * Department of Genetics, Howard Hughes Medical Institute, Harvard Medical School, Boston, Massachusetts 02115; † Division of Genetics, Brigham and Women’s Hospital, Boston, Massachusetts 02115

**Keywords:** lentivirus, genetic libraries, pooled shRNA or CRISPR screens, barcode screening, GC bias

## Abstract

Genetic screens are invaluable tools for dissection of biological phenomena. Optimization of such screens to enhance discovery of candidate genes and minimize false positives is thus a critical aim. Here, we report several sources of error common to pooled genetic screening techniques used in mammalian cell culture systems, and demonstrate methods to eliminate these errors. We find that reverse transcriptase-mediated recombination during retroviral replication can lead to uncoupling of molecular tags, such as DNA barcodes (BCs), from their associated library elements, leading to chimeric proviral genomes in which BCs are paired to incorrect ORFs, shRNAs, *etc*. This effect depends on the length of homologous sequence between unique elements, and can be minimized with careful vector design. Furthermore, we report that residual plasmid DNA from viral packaging procedures can contaminate transduced cells. These plasmids serve as additional copies of the PCR template during library amplification, resulting in substantial inaccuracies in measurement of initial reference populations for screen normalization. The overabundance of template in some samples causes an imbalance between PCR cycles of contaminated and uncontaminated samples, which results in a systematic artifactual depletion of GC-rich library elements. Elimination of contaminating plasmid DNA using the bacterial endonuclease Benzonase can restore faithful measurements of template abundance and minimize GC bias.

Genetic screening in cultured mammalian cells is a robust and effective means for discovering novel regulators of biological processes (Schlabach *et al.* 2008; Ashworth and Bernards 2010). Pooled genetic libraries are efficient tools for this purpose, and are more cost-effective for screening than arrayed libraries. Several streamlined, optimized protocols for pooled library screens have been published (Sims *et al.* 2011; Hoshiyama *et al.* 2012; Strezoska *et al.* 2012; Hu and Luo 2012); however, such experiments are typically plagued by high proportions of false positives (Stone *et al.* 2007; Mohr *et al.* 2010). This observation suggests that a ceiling of detection exists, perhaps owing to noise from systematic biases inherent in screen workflows. Here, we describe two sources of such noise and suggest methods to diminish their effects.

Pooled genetic screens typically involve transduction of cultured cells with retroviruses or lentiviruses encoding a genetic element of interest (shRNA, gRNA, ORF, *etc*.) (Cooray *et al.* 2012). These viral genomes become integrated into the host cell genomic DNA, enabling continuous, consistent expression of genetic elements over the course of many generations (Cooray *et al.* 2012). These stably transduced cells are then exposed to selective pressures, resulting in enrichment or depletion of the specific library elements that affect relevant biological pathways. These elements (screen hits) are identified by PCR-recovery of their encoding sequences or linked DNA barcodes (BCs) from the genomic DNA, and measurement of their abundance relative to a control population by microarray hybridization (Schlabach *et al.* 2008) or next-generation sequencing (Meyer and Kircher 2010; Hoshiyama *et al.* 2012).

Each step in this process represents an opportunity for the introduction of noise or error. For instance, retrovirus and lentivirus (a type of retrovirus) virions each carry two viral genomes (Paillart *et al.* 2004). During reverse transcription, the viral reverse transcriptase (RT) can stall or dissociate from one of these RNA genomes and reassociate with the other, resulting in template switching, which has the potential to generate a recombinant DNA provirus (Hu and Temin 1990; Smyth *et al.* 2012). For viruses encoding a single shRNA, gRNA, or ORF, such recombination will not be detectable in the final integrated proviral DNA. However, in the case of pooled genetic libraries containing thousands of unique elements, the two genomes inside a single virion will nearly always encode distinct library elements. We show that, in libraries using unique DNA BCs as reporters for library elements, RT-mediated recombination can result in chimeric proviral genomes that uncouple these library elements from their associated BCs. This renders BCs unfaithful and ineffective as reporters. We demonstrate that minimizing the distance between library elements and their associated BCs can substantially diminish this effect.

PCR recovery of library elements is also a critical step in pooled screening approaches, enabling sensitive and quantitative measurement of element abundance. However, PCR is known to be a source of bias in measuring abundance of multi-template populations (Kanagawa 2003; Kalle *et al.* 2014). Accurate, representative amplification of such populations requires careful optimization of cycle number and reaction conditions (Hoshiyama *et al.* 2012; Strezoska *et al.* 2012). We demonstrate that variation in template abundance between screen and control populations results in inaccuracies in fold-change measurements. A common source of such variation is the virus production method, in which excess plasmid is delivered to packaging cells during transfection, and is then carried over to target cells with viral supernatant during transduction (Segura *et al.* 2013). This excess plasmid dilutes out over time, but can remain intact in early samples of transduced cell populations used as the initial screen reference. This plasmid DNA will then be copurified with cellular genomic DNA, and can act as a template for PCR, sometimes leading to large differences in template abundance when compared with cell populations from the screen end-point, usually collected much later, after the plasmid has diluted away. A further consequence of these measurement inaccuracies is a bias toward enhanced recovery of GC-rich library elements from plasmid-contaminated samples, relative to their recovery from later time-point samples, due to a difference in effective number of PCR cycles. This difference leads to a perceived depletion of GC-rich templates. We demonstrate that the bacterial nuclease Benzonase can be used to degrade contaminating plasmid DNA from viral supernatant prior to transduction, minimizing plasmid carry-over, and eliminating GC-bias (Sastry *et al.* 2004).

## Materials and Methods

### Cell culture

U2OS osteosarcoma cells (obtained directly from American Type Culture Collection, HTB-96) were maintained in McCoy’s 5A medium supplemented with 10% (v/v) FBS (Hyclone), 100 units/ml of penicillin, and 0.1 mg/ml streptomycin. 293T cells were maintained in DMEM supplemented with 10% (v/v) FBS (Hyclone), 100 units/ml penicillin, and 0.1 mg/ml streptomycin. hTERT-immortalized Human Mammary Epithelial Cells (HMECs) from a reduction mammoplasty were purchased directly from Lonza (CC-2551), immortalized with human telomerase, and maintained in MEGM (Lonza).

### Virus production

In our standard lentivirus production protocol, 293T cells are seeded 24 hr before transfection at 1 × 10^7^ cells per 150 mm dish. For transfection, plasmid DNA is diluted into reduced-serum medium (Opti-MEM, Life Technologies) for a final volume of 3 ml; 24 µg each of target lentiviral plasmid and a lentiviral packaging plasmid mixture (1:1:1:1 of SV40 VSVg, Gag/pol, TAT, and Rev) are added to Opti-MEM, then 108 µl of TransIT-293 reagent (Mirus) is added to diluted DNA, mixed and incubated at room temperature for > 20 min. Where indicated, mass of plasmid DNA used for transfection was reduced to 10% or 20% relative to the standard protocol. The volume of TransIT-293 is always equal to 3× the total mass of plasmid DNA in the mixture. Following incubation, resulting lipid complexes are added dropwise to cells. After 14–18 hr, transfection medium is removed and replaced with 15–20 ml of normal culture medium. At 24–36 hr following medium replacement, viral supernatants are collected, aliquoted and stored at –80° until further use. See Supplemental Material, File S1 for further information.

### Benzonase treatment

Lentiviral supernatants were treated with Benzonase as described (Sastry *et al.* 2004), using 100–1000 units/ml of viral supernatant, and 10× Benzonase Buffer (500 mM Tris-HCl, pH 8.0, 10 mM MgCl_2_, and 1 mg/ml bovine serum albumin) at a final concentration of 1×. The mixture was incubated for 30 min at 37° following addition of Benzonase. Lentivirus was then aliquoted and stored at –80° until use. For data in Figure 3A, a 3′BC library virus was treated with 50 units/ml Benzonase for 15 min at 37°. The Benzonase was inactivated by treatment with 10 mg/ml proteinase K for 1 hr at 55°. Proteinase K was inactivated by incubating samples at 98° for 20 min. See Supplemental Material, File S1 for further information.

### Virus titration

Lentiviral titers were determined by transducing U2OS cells with serial dilutions of virus by adding diluted viral supernatant directly to culture medium supplemented with 4 μg/ml polybrene. At 16 hr after transduction, culture medium was replaced. At 2 d after transduction, 1.5 µg/ml Puromycin (Clontech) was added to culture medium and replaced every 3 d. Cells were allowed to grow until visible colonies formed. Medium was then removed and cells were stained with 0.5% Methylene Blue in 50% Ethanol for 30–60 sec. Colonies were counted and used to determine titer based on the volume of virus used.

### RT-qPCR

Quantitative RT-PCR (RT-qPCR) was performed in triplicate using the Platinum Sybr Green Kit (Invitrogen) on an Applied Biosystems Fast 7500 machine. Detection of *CCND1* ORF abundance was performed using Relative Quantitation (RQ) settings, and was normalized to the total integrated ORF library using primers targeting the PGK promoter as the endogenous control (*CCND1* Forward: 5′ GCTGTGCATCTACACCGACA 3′; Reverse: 5′ TTGAGCTTGTTCACCAGGAG 3′. PGK Forward: 5′ CAA CCG GCT CCG TTC TTT GG 3′; Reverse: 5′ CAC GAG ACT AGT GAG ACG TGC TA 3′). Detection of combined genomic DNA (gDNA) and plasmid signal using CMV or PGK primers was performed using RQ settings, and was normalized to total gDNA using primers targeting the *GAPDH* promoter as the endogenous control (CMV Forward: 5′ TGGCATTATGCCCAGTACATGACC 3′; Reverse: 5′ CCATTGATGTACTGCCAAAACCGC 3′. *GAPDH* Forward: 5′ ATC CAA GCG TGT AAG GGT CC 3′; Reverse: 5′ GGA CTG AGA TTG GCC CGA TG 3′). Detection of shRNA half-hairpins was performed in this same manner, using primers JH353F (5′ TAGTGAAGCCACAGATGTA 3′) and HHR2L (5′ ATGTATCAAAGAGATAGCAAGGTATTCAG 3′), with *GAPDH* promoter as the endogenous normalization control.

### Screens

HMECs were transduced with library lentivirus in triplicate with 8 µg/ml polybrene (Sigma), with an average representation of 1000 cells per ORF using a multiplicity of infection (MOI) of 0.5. Medium was changed the following day. Cells were split 2 d following transduction, and a portion equivalent to ≥ 1000× representation was collected from each replicate and stored at –80° to use as a reference. Sufficient cells to maintain ≥ 1000× representation were then cultured for 10 population doublings (PDs) [following the first split, stably transduced cells were selected with 2 µg/ml Puromycin (Clontech) for 3–4 d], and final cell pellets containing ≥ 1000× library coverage were collected.

For screens performed using the inducible tetracycline responsive element (TRE) BC library, transduced cells were fully selected with Puromycin before induction. At the time of induction, a portion of cells equivalent to ≥ 1000× representation was collected from each replicate and stored at –80° to use as a reference. Sufficient cells to maintain ≥ 1000× representation were then cultured in the presence or absence of doxycycline (dox) at 100 ng/ml for 10 PDs, when final cell pellets were collected. The shRNA screen depicted in Figure 4A was performed as described (Schlabach *et al.* 2008). The mock screen described in Figure 4, C–D was performed using U2OS cells, infected at MOI < 0.5 in duplicate with 4 µg/ml polybrene (Sigma). Medium was replaced 24 hr after transduction. Initial normalization samples were harvested 3 d after transduction, and cells were then selected with Puromycin (1.5 µg/ml). Following selection, cells were seeded in the presence or absence of 1 µg/ml dox. Library representation of an average of 200 copies of each shRNA was maintained at every passage. Final screen samples were harvested after 10 PDs in the presence (or absence) of dox.

### Genomic DNA preparation and PCR

Screen cell pellets were thawed and lysed in 10 mM Tris, pH 8.0, 10 mM EDTA, 0.5% SDS, 0.75 mg/ml Proteinase K at 55° overnight. gDNA was extracted using Phase Lock tubes (5 PRIME) with phenol:chloroform and then chloroform. RNase A was added to a final concentration of 25 μg/ml and, following incubation overnight at 37°, gDNA was again extracted using Phase Lock tubes with phenol:chloroform, followed by two chloroform extractions. DNA was ethanol precipitated, recovered by centrifugation, and washed three times with 70% ethanol. Dried pellets were resuspended in 10 mM Tris-Cl, pH 8.5, and BCs were PCR-amplified with Phusion High-Fidelity DNA Polymerase (NEB Cat # M0530S) in three PCR steps for BC recovery, addition of Illumina adaptors, and sample indexing (Meyer and Kircher 2010). The first round of PCR was performed using common primers flanking the unique BC region (forward primer ORF.BC1.for: 5′ CCAGTAGGTCCACTATGAGT 3′; reverse primer ORF.BC.1.rev: 5′ CTAGTTCCGCTTACACAGCT 3′). The reaction contained a total mass of DNA equal to gDNA from cells covering 1000× representation, with individual 100 µl reactions containing 10 µg gDNA, 1× Phusion HF Buffer, 200 µM dNTPs, 1 µM each of ORF.BC1.for and ORF.BC1.rev, and 4 units of Phusion Polymerase. Reactions for each replicate were pooled, and 5 µl of pooled PCR1 was used as the template for a 100 µl reaction containing 1× Phusion HF Buffer, 250 µM dNTPs, 2 µM each of primers ISP-ORF.BC1.for (5′ GTGACTGGAGTTCAGACGTGTGCTCT TCCGATCTCCAGTAGGTCCACTATGAGT 3′) and P5-ORF.BC1.rev (5′ AATGATACGGCGACCACCGACTAGTTCCGCTTACACAGCT 3′), and 4 units of Phusion Polymerase. A third PCR reaction was performed to add indices and allow sample multiplexing. The 100 µl PCR3 reaction contained 2 µl of PCR2 product, 1× Phusion HF Buffer, 250 µM dNTPs, 2 µM each of primers P7-Index-ISP (Meyer and Kircher 2010) and P5-ORF.BC1.rev (5′ AATGATACGGCGACCACCGACTAGTTCCGCTTACACAGCT 3′), and 4 units of Phusion Polymerase. PCR3 products were gel-purified using QiaQuick Gel Extraction columns (Qiagen). Samples were sequenced on two Illumina HiSequation 2500 lanes with the primer heyME19 (5′ GCGACCACCGACTAGTTCCGCTTACACAGCT 3′).

For the mock shRNA screens, half-hairpin amplicons were recovered by PCR using primers JH353F (5′ TAGTGAAGCCACAGATGTA 3′) and HHR2L (5′ ATGTATCAAAGAGATAGCAAGGTATTCAG 3′). Amplified half-hairpins were gel-purified, and amplified by two additional rounds of PCR to add Ion Torrent adapter sequences, and sample index sequences. Final PCR products were gel purified and prepared for Ion PGM sequencing using the Ion PGM Template OT2 200 Kit (Life Technologies) according to manufacturer’s instructions. Sequencing was performed using the Ion PGM Sequencing 200 Kit v2, and the Ion 318 Chip Kit v2, according to manufacturer’s instructions.

### PCR assay to detect ORF-BC recombination

For PCR assays to measure the frequency of ORF-BC recombination, 11 clones were picked at random from each ORF-BC library, and sequenced to identify individual ORF-BC pairs. Clone plasmid DNA was transiently transfected into 293Ts individually, or pooled together for transfection, to produce lentivirus. hTERT-HMECs were transduced with individual or pooled lentivirus, and stable integrants were selected with Puromycin (2 µg/ml). gDNA was isolated from each stable cell line. PCR using primers targeting one of the 11 ORFs and a common region of the vector (5′ BC Recombination Test: 5pBC.KRTAP19-7.ORF: 5′ CCACATCCACAGCTATAGC 3′; ORF.BC1.for: 5′ CCAGTAGGTCCACTATGAGT 3′. 3′ BC Recombination Test: 3pBC.MAX.ORF: 5′ CAACCGAGGTTTCAATCTGC 3′; ORF.BC1.rev: 5′ CTAGTTCCGCTTACACAGCT 3′) were used to amplify BCs from either the pooled lentivirus-transduced gDNA, or the combined gDNA from each of the individual lentivirus-transduced cells. PCR products were cloned using the pENTR/D-TOPO Cloning Kit (Life Tech K2400-20), and clones were sequenced to determine BC identity.

### Data availability

The authors state that all data necessary for confirming the conclusions presented in the article are represented fully within the article.

## Results

### Barcode recombination causes reporter infidelity

**Figure 1:**
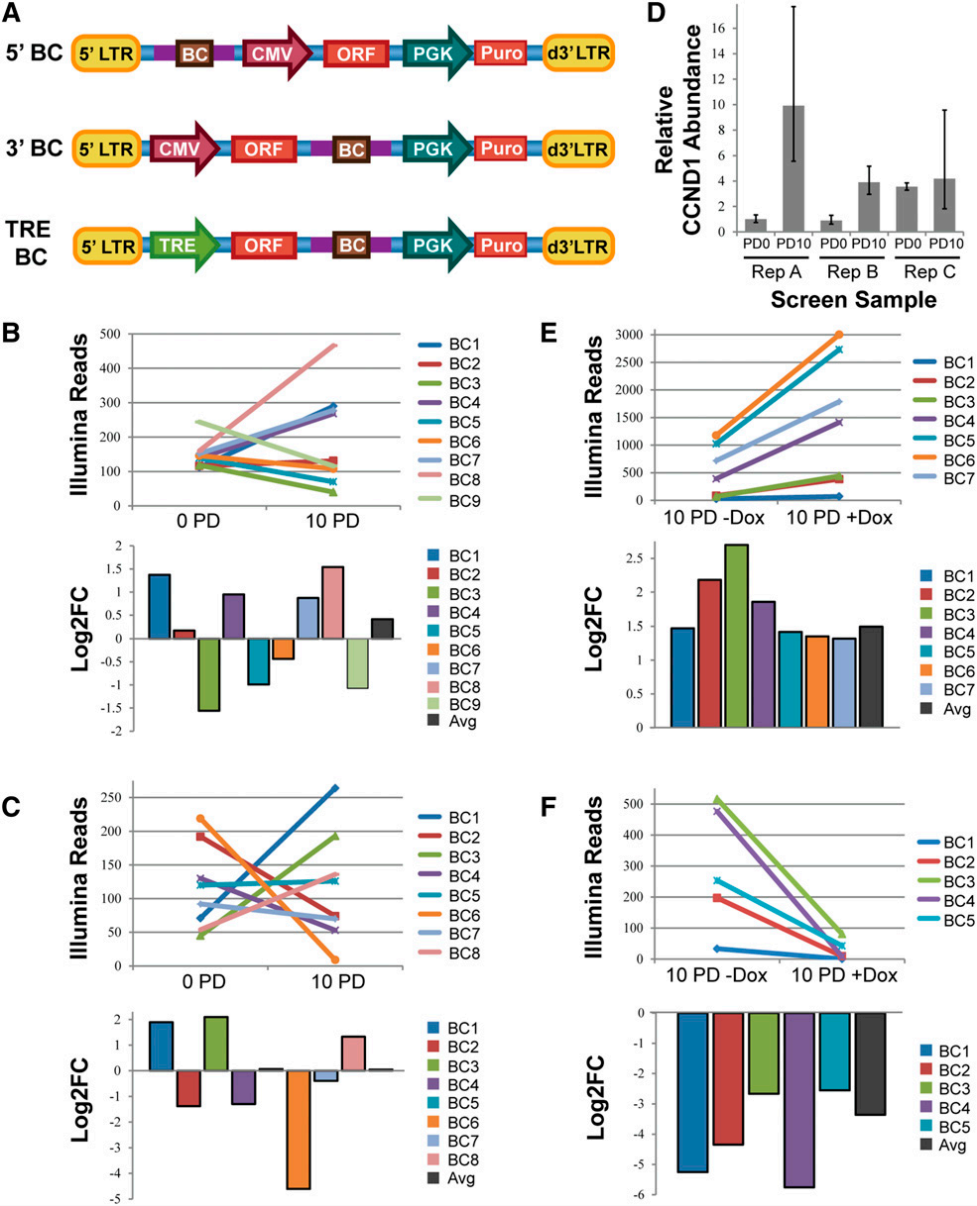
Barcode agreement in 5′ BC and TRE BC libraries. (A) Vector schematics of three library vectors constructed in the pHAGE lentiviral backbone. Note that the TRE BC Library was used in cells that had previously been transduced with a lentivirus carrying the reverse tetracycline transactivator (rtTA). LTR, long terminal repeat; BC, barcode; CMV, cytomegalovirus immediate-early promoter; PGK, phosphoglycerate kinase 1 promoter; TRE, tetracycline responsive element promoter. (B, C) Distribution of normalized read counts in initial (0 PD) and final (10 PD) screen samples (top panel) and Log2FC (bottom panel) for a representative set of BCs mapped to *CCND1* (B) or *CDKN1A* (C) in the 5′BC library. (D) RT-qPCR measurement of *CCND1* abundance in gDNA from three replicates measured at initial (PD0) and final (PD10) time points of the 5′BC library screen. Assays were performed in triplicate (error bars ± SD). (E, F) Distribution of normalized read counts in initial (10 PD –Dox) and final (10 PD +Dox) screen samples (top panel) and Log2FC (bottom panel) for a representative set of BCs mapped to *CCND1* (E) or *CDKN1A* (F) in the TRE BC library.

To enable high-throughput gain-of-function screens, we recently designed human ORF libraries in which each ORF is paired with unique DNA BCs (Ho *et al.* 2009; Yang *et al.* 2011; manuscript in preparation). The uniform length of the BCs (30 bp) enables more quantitative PCR recovery, as it alleviates the inherent bias introduced by amplification of templates that vary in length (Shagin *et al.* 1999). Furthermore, our barcoding strategy facilitates the pairing of each ORF with several independent BCs providing a measure of internal reproducibility. We designed a flexible, modular lentiviral vector for library expression and tested several iterations in which we varied the position of the BC and the promoter (Figure 1A). We performed identical proliferation screens in HMECs with each version of this library, identifying BCs that shifted in abundance over 10 PDs.

Surprisingly, we frequently observed discordance in the log_2_ of the fold-change ratios (Log_2_FC) among the sets of BCs reporting for the same ORF in the 5′BC library. While neutral genes might be subject to random drift, causing some BCs to become slightly enriched, and others slightly depleted, we observed marked divergence of BC abundance changes among BCs paired with ORFs known to potently regulate proliferation, such as *CCND1* and *CDKN1A* (Figure 1, B and C). Furthermore, when we measured the abundance of the *CCND1* ORF in the 5′BC screen gDNA by qPCR, we saw that the ORF was becoming enriched over time in at least two of the three replicates (Figure 1D), though this was not reflected by the average behavior of the paired BCs (Figure 1B). We sought to investigate and correct the source of this variation, as the fidelity of our BCs as specific and precise reporters for ORF behavior is critical to the success of our library design.

One possible explanation for this observation was retrovirus-mediated recombination between BC-ORF pairs causing shuffling of these library elements, because retroviruses carry two copies of their genome and reverse transcribe them with a recombination-prone RT enzyme (Paillart *et al.* 2004; Smyth *et al.* 2012). To test whether retroviral-mediated recombination occurred in our libraries, and at what frequency, we assembled small sublibraries containing 11 ORF clones from the 5′BC or the 3′BC libraries, each associated with a single, unique, BC. Plasmid DNA containing a mixture of all 11 clones was used to package lentivirus for HMEC transduction. We isolated gDNA from stably transduced cells, and then designed PCR primers to amplify the BCs attached to one of the 11 ORFs using an ORF-specific primer, and a common primer that anneals to the vector. The final PCR product contains the associated BC. We then sequenced these PCR products to identify the associated BCs.

One concern in using PCR to amplify proviral integrants for measurement of recombination frequency is the possibility that PCR artifacts could distort this measurement. Specifically, the PCR reaction itself can generate chimeric products, which, for our purposes, could lead to perceived recombination between ORFs and BCs (Brakenhoff *et al.* 1991; Suzuki and Giovannoni 1996). To control for this possibility, we also prepared lentiviruses from each of the 11 BC-ORF clones from the two sublibraries individually. We transduced cells with these individual lentiviruses, selected stable integrants, and isolated gDNA. We then pooled gDNA from each cell line infected with a single individual lentivirus together into a single PCR reaction. Thus, both our PCR control and recombination test conditions consisted of a population of heterogeneously infected cells, each carrying a single integrant of one of 11 possible ORF clones. However, only in the recombination test condition could retrovirus-mediated recombination contribute to any observed shuffling of BCs between ORFs.

We observed recombination events in > 25% of the sequences tested from the 5′BC library (Table 1). Conversely, in the 3′BC library, recombination occurred at a measured frequency of only 5.5%. This substantial difference in recombination frequency (*P* = 0.00015, binomial test) correlates with the length of homologous sequence residing between the ORF and the BC (720 bp in the 5′BC library, and 96 bp in the 3′BC library), consistent with previous reports (Delviks and Pathak 1999; Hu *et al.* 2003). Importantly, no recombination was observed in our PCR control conditions, indicating that the retroviral reverse transcription step is the likely source of this BC-ORF uncoupling. It should be noted that our measurements will underestimate the true recombination frequency, because we are unable to detect recombination events that occur between identical viruses within our pool. As this is expected to occur in 9% of cases for a sublibrary of 11 ORFs, we estimate the true recombination frequency to be greater than 28% in the 5′BC library, and ∼ 6% in the 3′BC library (Table 1). Screens performed using the TRE BC library, in which the ORF-BC pairs are identical to those in the 3′BC library and only the promoter is varied, exhibit strong concordance among BCs paired with ORFs known to promote strong proliferation phenotypes, such as *CCND1* and *CDKN1A* (Figure 1, E and F).

**Table 1:** Frequency of retroviral-mediated recombination in BC-ORF libraries

Library	Distance Between ORF and BC (bp)	Experimental Condition	Number of Sequences Analyzed	Number of Recombined BCs	Measured Frequency	Estimated True Frequency*^a^*
5′BC	720	Recombination test	65	17	26.15%	28.53%
5′BC	720	PCR control	33	0	0%	0%
3′BC	96	Recombination test	55	3	5.45%	5.95%
3′BC	96	PCR control	20	0	0%	0%

Results of the recombination test performed using sublibrary of 11 ORFs. The BCs paired to selected ORFs are recovered by ORF-specific PCR and identified by Sanger sequencing.

a Sum of the measured frequency and the calculated frequency of undetected recombination events in homozygous virions, predicted to be ∼ 9.09% of the total population of virions.

### Plasmid contamination of viral supernatants distorts distributions of BC abundance

**Figure 2:**
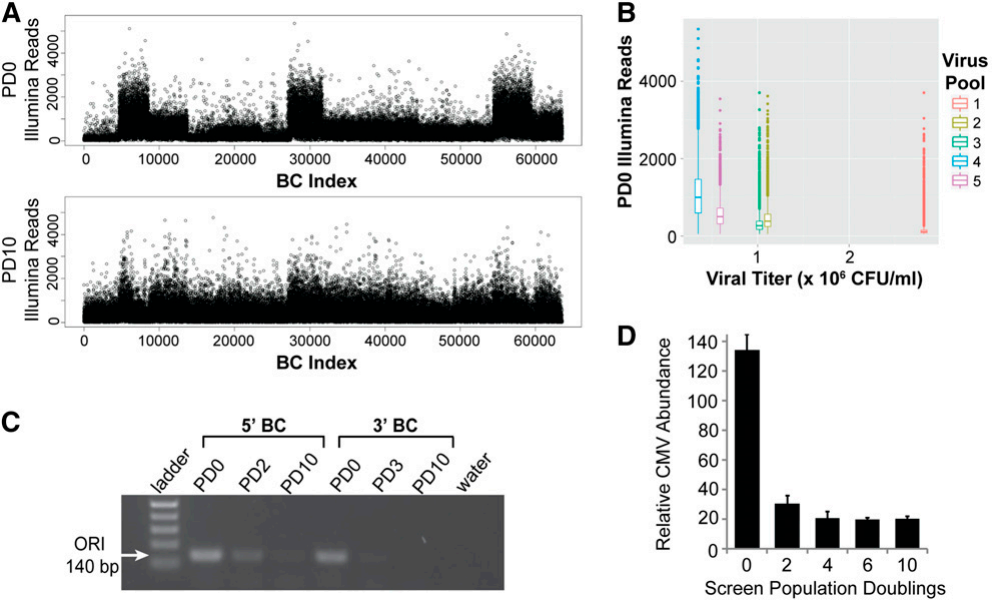
Plasmid DNA contaminates lentiviral supernatants and is detected in screen gDNA. (A) Distribution of Illumina reads per BC from the 3′BC Library screen at 0 PD (top panel) and 10 PD (bottom panel). (B) Barplot distribution of reads per BC at 0 PDs *vs.* viral titer in colony forming units per milliliter (CFU/ml) for each of five subpools of lentivirus that were coinfected at equal viral representation for full 3′BC library coverage. (C) Agarose gel showing PCR product from indicated screen gDNA sample using primers specific for the bacterial origin of replication (ORI). (D) RT-qPCR measurements of relative CMV abundance at indicated screen time-points in 5′BC library cells as a measure of the combined signal of library virus-infected cells and contaminating plasmid within gDNA. Assays were performed in triplicate (error bars ± SD).

The 3′ and 5′BC libraries were constructed of five or 13 distinct pools of ORF clones, respectively. Each pool was packaged into lentivirus and titered individually, and these were then combined for transduction and pooled screening of the complete collection. In the context of constitutive expression, we sought to maximize the dynamic range of these screens by collecting initial reference samples of cells 2 d after transduction with library virus. Additional samples of cells were collected at each passage, and a final sample was taken after 10 PDs. In both of these screens, deep sequencing of initial reference samples revealed a nonrandom distribution of read density across the library BCs (Figure 2A and Table S1). Subpopulations of BCs were present at substantially (2- to 10-fold) different abundances. Furthermore, these differences did not persist at 10 PDs, suggesting a transient effect (Figure 2A). The subpopulations corresponded to our library lentivirus pools, and the signal abundance of each pool inversely correlated with its viral titer (Figure 2B). This suggested that the source of overrepresentation of individual pools may have been the lentivirus itself, since a larger volume of the lower titer viral supernatants was added to cells to normalize the representation of different pools.

We hypothesized that, following transfection for virus production, residual plasmid DNA may remain in the viral supernatant, and be carried over to the target cells at the time of transduction. To test this hypothesis, we asked whether we could detect evidence of plasmid DNA in the gDNA isolated from our screen cell samples. We designed primers annealing to the bacterial origin of replication (ORI), which is absent in the integrated viral DNA, and observed a PCR product of the correct size (Figure 2C). In samples from both the 3′ and 5′BC libraries, band strength in each sample roughly corresponded to the observed pattern of distortion in Illumina reads for that time-point. We reasoned that, using primers targeting a portion of the lentiviral genome that integrates into cells, we could detect the combined signal from both plasmid and proviral integrations in each screen sample. Using RT-qPCR primers to amplify the CMV promoter region of our vector, we confirmed the presence of a large overabundance of signal at the initial time-point of the 5′BC screen, most of which disappeared after 2 PDs, leaving a smaller population of plasmid which appears to dilute out completely by 6 PDs (Figure 2D). Presumably, the remaining signal is generated only by proviral integrants, as the contaminating plasmid is no longer present. We estimate the amount of plasmid DNA in the initial 5′ BC screen samples to be ∼ 5.8 pg per 50 ng of total extracted DNA, corresponding to > 70 times more copies of the viral genome than are present integrated into the genomic DNA.

### Elimination of plasmid DNA from viral supernatant

**Figure 3:**
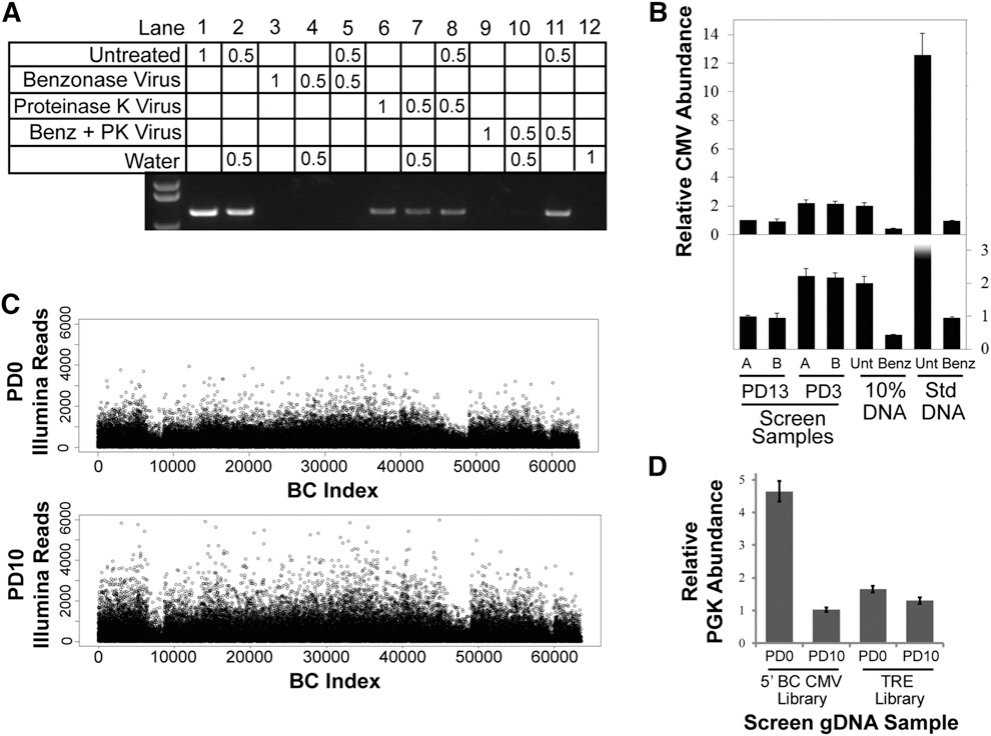
Elimination of plasmid DNA from lentivirus by treatment with Benzonase. (A) Agarose gel showing PCR products from bacterial-derived portion of plasmid DNA in library viral supernatants. Lane labels denote the volume of indicated template used in each PCR reaction in microliters. Benzonase Virus, viral supernatant treated with Benzonase; Proteinase K Virus, viral supernatant treated with Proteinase K; Benz + PK Virus, viral supernatant treated with Benzonase, followed by treatment with Proteinase K. (B) RT-qPCR measurements of relative CMV abundance as a measure of the combined signal of library-virus-infected cells, and contaminating plasmid within gDNA. Cells were infected at MOI = 0.5 with virus prepared according to our standard lentiviral production protocol (Std DNA), or by reducing the amount of transfected plasmid DNA to 10% of that in our standard protocol (10% DNA). These viruses were then either treated with Benzonase (Benz) or left untreated (Unt), and were used to transduce HMECs. Genomic DNA was isolated 3 d after transduction, and CMV abundance was compared to gDNA from two of three replicates (A and B) of the 3′BC screen at 3 PD and 13 PD. The top panel shows full data range, and the bottom panel shows an expanded view of lower abundance samples. Assays were performed in triplicate (error bars ± SD). (C) Distribution of Illumina reads per BC from the inducible TRE BC library at 0 PD (top panel) and 10 PD (bottom panel). Library virus was treated with 500 units/ml Benzonase before transduction. (D) RT-qPCR measurements of relative PGK abundance as a measure of the combined signal of library-virus-infected cells and contaminating plasmid within gDNA. The TRE BC library lentivirus supernatant was treated with Benzonase and transduced into HMECs which were passaged for 6 PDs in the absence of doxycycline before collecting initial PD0 time point. PGK abundance from the inducible TRE library samples was compared to samples from the 5′BC Library screen. Assays were performed in triplicate (error bars ± SD).

We observed that decreasing the amount of plasmid DNA used in our transfection protocol reduced levels of contaminating plasmid, without substantially diminishing viral titers (Figure 3B). However, even a 10-fold reduction in input DNA failed to generate transduced cell populations completely free of contamination. Previous work has demonstrated that the bacterial nuclease Benzonase is effective for eliminating plasmid contamination from clinical preparations of lentivirus (Sastry *et al.* 2004). This endonuclease can degrade both DNA and RNA, so contaminating plasmid DNA is accessible for degradation, while enveloped viruses are protected. Indeed, Benzonase treatment is commonly included in the workflow for large-scale lentivirus vector production for gene therapy applications to avoid the immunogenic consequences of naked DNA (Segura *et al.* 2013).

We thus tested whether Benzonase treatment could eliminate detectable levels of plasmid DNA from viral supernatants. As Benzonase is highly stable, and retains activity even after storage at –80°, any PCR-based method for detection of residual plasmid in Benzonase-treated virus is masked by degradation of PCR products (Figure 3A). To visualize PCR products and assess plasmid levels in our virus, we needed to inactivate Benzonase without affecting the subsequent PCR reaction. We found that the nonspecific serine protease Proteinase K could completely digest Benzonase, so that no nuclease activity remains (Figure 3A). Conveniently, Proteinase K can be heat-inactivated, allowing the PCR reaction to proceed uninhibited. While Benzonase-treated virus without subsequent inactivation masks the signal of spiked-in untreated virus (Figure 3A, lane 5), use of Proteinase K to inactivate Benzonase following virus treatment yields apparent elimination of plasmid (lanes 9–10) but no longer suppresses the signal of spiked-in untreated virus (lane 11).

In the context of genetic screens, our greatest concern was to ensure that plasmid contamination was no longer present at the time of collecting samples of transduced cells for screen reference samples. To test this, we packaged lentivirus using our standard transfection protocol or using one-tenth of the amount of plasmid in our standard protocol. These lentiviruses were then treated with Benzonase or left untreated. We infected HMECs at MOI < 0.5, as we had done for our screens. Cells were harvested 3 d after transduction, gDNA isolated, and residual plasmid levels were detected by qPCR of the CMV promoter. In comparing our test samples to gDNA from 3′BC library screen cells that had undergone 13 PDs, and no longer exhibited any distortion of sequence reads due to plasmid contamination, we identified a baseline abundance of CMV that corresponds to a single proviral integration per diploid genome (Figure 3B). As expected, gDNA from 3′BC library screen cells that had undergone only 3 PDs after transduction exhibited about twice as much CMV signal, half of which must derive from plasmid DNA. While our untreated viruses exhibit contamination levels that correspond to the relative amount of transfected DNA, Benzonase treatment substantially reduces detection of residual plasmid. Notably, only when input DNA was reduced to 10% of standard concentrations were we able to see CMV abundance reflective of the MOI of 0.5, suggesting complete elimination of contaminating plasmid (Figure 3B) (the screen samples shown for comparison are all derived from cells that had been selected for integrants, thus would correspond to an MOI ≥ 1).

For screens using the TRE BC libraries, library virus was generated by transfecting a reduced amount of input plasmid DNA (20%) relative to our standard protocols and viral supernatants were treated with Benzonase. Library-infected cells were then passaged for several PDs in the absence of doxycycline (dox), before initial reference samples were collected, and dox was added to induce ORF expression. We compared contamination levels of our TRE screen gDNA samples to samples from the 5′BC library, using primers amplifying the PGK promoter that drives expression of the Puromycin resistance gene in both vectors (Figure 1A). We observe nearly complete elimination of plasmid at PD0 (Figure 3D). As expected, in the absence of plasmid contamination, the distributions of Illumina reads at 0 PD and 10 PD are nearly identical (Figure 3C and Table S2), with the persistent visible variations in abundance between viral subpools likely corresponding to real differences in MOI resulting from imperfect estimates of viral titer.

### GC bias upon PCR amplification

Several aspects of multi-template PCR reactions can contribute to variation in amplification efficiencies based on the GC richness of each template. While we generally observe less efficient recovery of high-GC elements, this effect in itself should not impact the computed fold-change values, as differences in efficiency should be uniform across screen samples. However, contaminating plasmid DNA present in screen genomic DNA may lead to different degrees of GC bias in different screen samples due to a different effective number of cycles of PCR in samples where the template is over-represented. Contaminated initial reference samples will experience fewer effective PCR cycles before the PCR reaction is depleted of primer or nucleotides than uncontaminated samples from later time-points. Thus, the overabundance of target DNA in normalizing initial reference samples would cause higher GC-containing elements to erroneously appear to be depleting from the screens, although this effect would be due to artifact rather than phenotype.

**Figure 4:**
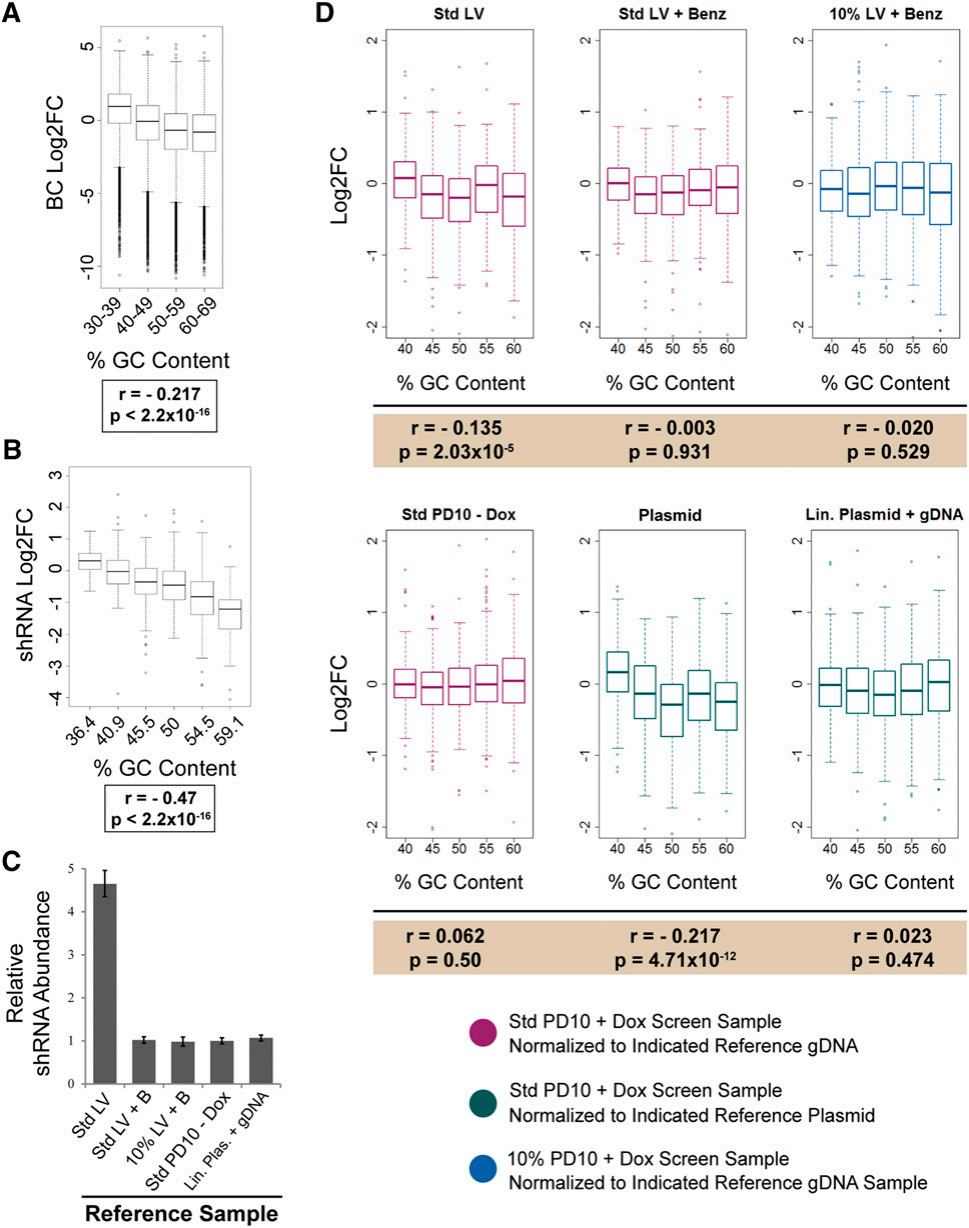
Variation in template abundance causes artificial depletion of high GC content library elements during screens. (A, B) Boxplot representation of the distribution of BC Log2FC (A) or shRNA Log2FC (B) binned by GC content. Median, first quartile, third quartile, and outliers (points) are shown. Data in (A) are from 5′BC library screen (Table S3). Data in (B) are from a viability screen performed using an shRNA library (Table S4). Only nontargeting negative control shRNAs are included. Pearson’s product-moment correlation coefficient (*r*) and *P*-value for correlation are indicated below each plot. (C) Relative shRNA abundance of reference samples used for mock screen normalization. shRNA abundance was measured by RT-qPCR and normalized to total gDNA. Assays were performed in triplicate (error bars ± SD). Std LV, lentivirus prepared according to standard protocol; Std LV + B, lentivirus prepared according to standard protocol and treated with Benzonase; 10% LV + B, lentivirus prepared using 10% of standard input plasmid DNA and treated with Benzonase; Std PD10 – Dox, cells infected with Std LV that have been passaged for 10 PDs in the absence of doxycycline; Lin. Plas. + gDNA, library plasmid DNA was linearized and mixed into genomic DNA from uninfected U2OS cells at a molar ratio equivalent to one virus insertion per diploid genome. (D) Boxplot representation of the distribution of mock screen shRNA Log2FC binned by GC content. Median, first quartile, third quartile and outliers (points) are shown. Plot colors correspond to screen and normalization sample combinations indicated. Pearson’s product-moment correlation coefficient (*r*) and *P*-value for correlation are indicated below each plot. Std LV, lentivirus prepared according to standard protocol; Std LV + B, lentivirus prepared according to standard protocol and treated with Benzonase; 10% LV + B, lentivirus prepared using 10% of standard input plasmid DNA and treated with Benzonase; Std PD10 – Dox, cells infected with Std LV that have been passaged for 10 PDs in the absence of doxycycline; Plasmid, library plasmid DNA used at an equivalent mass to gDNA samples; Lin. Plas. + gDNA, library plasmid DNA was linearized and mixed into genomic DNA from uninfected U2OS cells at a molar ratio equivalent to one virus insertion per diploid genome.

We examined the relationship between GC content of BCs and their Log_2_FC in our 5′BC and 3′BC library screens, and indeed observed a correlation (Figure 4A and Table S3). We then asked whether this effect extended to other types of library elements, and examined a set of nontargeting shRNAs carried through a viability screen for 10 PDs as internal negative controls. Again, a correlation was observed between the GC content of each hairpin and its dropout during the screen leading to a two- to three-fold depletion of GC rich sequences (Figure 4B and Table S4).

To test whether elimination of plasmid contamination could abolish the observed GC bias, we performed a mock screen using a library of 1000 nontargeting shRNAs. The library contains five pools, each with 200 shRNAs of uniform GC content: 40%, 45%, 50%, 55%, or 60% GC bases in the hairpin sequence. The shRNAs were expressed from a Tetracycline-inducible lentiviral vector. We pooled these plasmid libraries together, and packaged them into viruses, either according to our standard protocol or following modified protocols for plasmid elimination, utilizing reduced plasmid DNA input and/or treating lentiviral supernatants with Benzonase. U2OS cells were infected with these viruses at an MOI < 0.5, and cells were collected 3 d posttransduction, and again after 10 PDs in the presence or absence of dox. We isolated gDNA, and recovered half-hairpin amplicons by PCR (Schlabach *et al.* 2008). The abundance of each shRNA in each of our screen samples and reference samples was quantified by deep sequencing of the half-hairpin amplicons.

To further assess the impact of plasmid DNA on relative PCR efficiency of GC-rich templates, we designed several additional reference samples. Rather than computing the fold-change of a hairpin relative to its abundance in infected cells, we assessed the direct quantification of plasmid DNA for this purpose. Some laboratories use library plasmid DNA, rather than infected cells to normalize screen data (Sims *et al.* 2011; Cheung *et al.* 2011). We found that the amount and structure of the plasmid DNA used as reference is important. It is critical to match the total number of template molecules in the gDNA and plasmid samples. In order to try to more precisely recreate the conditions of PCR in our screen samples, we also linearized library plasmid DNA, and mixed it with untransduced gDNA from U2OS cells, so that the number of template copies was equivalent to library-infected cells, and both contain equivalent amounts of nontemplate, burden DNA. This reference condition also mimics the structure of the template DNA molecules in screen sample PCRs, which are the relaxed rather than the supercoiled form of plasmid DNA.

We measured the abundance of the shRNA half-hairpin template in each of our reference conditions by qPCR (Figure 4C). Though the baseline level of contamination was modest, we observed a statistically significant correlation between GC content and Log_2_FC when cells infected with lentivirus produced under standard conditions were used as the reference sample for normalization (Figure 4D, top left panel, and Table S5). In contrast, reducing the amount of DNA used for transfection, or treating lentiviral supernatants with Benzonase, eliminated this correlation. Additionally, normalizing screen samples to cells which had been carried for 10 PD in the absence of dox induction also successfully eliminated observed GC bias. Though infected with contaminated lentivirus, these cells underwent sufficient doublings for dilution of the plasmid to occur, allowing the population to reach equilibrium. While we observed a significant negative correlation between GC richness and Log_2_FC when plasmid DNA was used as the reference (without matching the number of template molecules to the gDNA screen sample) (Figure 4D, bottom center panel), we found that normalizing our screen samples to a reference sample consisting of diluted linearized plasmid DNA was effective in eliminating GC bias. Importantly, shRNA half-hairpin template was over 200-fold more abundant in the plasmid sample relative to the linearized-plasmid in gDNA condition. This suggests that the total number of copies of the template present in the PCR reaction can dramatically influence the perceived relative abundance of GC-rich targets.

## Discussion

In this study, we report several sources of error intrinsic to common genetic screening techniques in mammalian tissue culture systems. For each, we suggest methods to eliminate or minimize them, which should result in improved fidelity of screen data. Most genetic screens in human cells generate sets of candidate genes which putatively play a role in the phenotype of interest, but these are plagued by a high proportion of false positives (Stone *et al.* 2007; Mohr *et al.* 2010). This ceiling of detection of true hits is often attributed to quality of the genetic element; for instance, variation in reagent knock-down efficiency and off-target effects are thought to account for many of the false-positives in candidate gene lists derived from RNAi screens (Echeverri *et al.* 2006; Hu and Luo 2012). We find that additional noise is introduced due to unrepresentative abundance measurements and GC bias attributable to variable library template concentration between reference and experimental samples during PCR amplification.

We find a common cause of variable library template representation to be the presence of contaminating plasmid DNA in cell populations shortly after transduction. Plasmid DNA remains in the viral supernatant following the transient transfection step required to produce lentivirus, and is transferred to infected cells along with the viruses. To minimize the effects of this contamination, we treated viral supernatants with the endonuclease Benzonase to degrade residual plasmid DNA. This method is well established in preparation of clinical-grade lentivirus for gene therapy applications (Sastry *et al.* 2004; Yang *et al.* 2012; Segura *et al.* 2013); however, to our knowledge, it has not been previously reported to be used in preparation of lentivirus for genetic screens or other tissue culture applications. We demonstrate that Benzonase treatment can significantly increase the fidelity of specific detection of integrated viral genomes from the genomic DNA of a population of infected cells. Furthermore, Benzonase treatment, or use of an inducible expression system passaged to equilibrium minimize the GC bias caused by plasmid contamination. This fidelity is critical to measurements of changes in abundance within the cell population through the course of a genetic screen for identification of screen hits.

Many genetic screens are normalized to the distribution of genetic elements present in the plasmid DNA library, rather than using a prescreen sample of the transduced cell population as a reference. In fact, a recent analysis suggested that plasmid DNA was the optimal reference point for shRNA screens, compared with viral cDNA or early time points immediately following viral integration (Sims *et al.* 2011). However, for the use of ORFs, this option would fail to report information about significant differences in lentiviral packaging efficiency correlated with transgene size, and thus would lead to the potential for overestimating the abundance of long ORFs, and underestimating the abundance of short ORFs (Kumar *et al.* 2001). Pooled shRNA or CRISPR-based libraries are uniform in size, and thus may be more amenable to use of plasmid as the reference for normalization. However, we find that care must be taken when using plasmid DNA as described below.

An additional effect of plasmid contamination is a persistent correlation between GC-richness of individual library PCR templates, and the dropout of those sequences under screening conditions. Two factors contribute to this effect. The first is the bias against amplifying GC rich templates in each PCR cycle (Figure 4, A and B). In principle, this should cancel out when two samples (*i.e.*, start and finish) are experiencing the same number of PCR cycles. The second is the possible difference in the total number of template molecules in the initial and end-point samples, which can be influenced by plasmid contamination during the initial transduction process. With a large increase in template molecules in the initial reference sample, those samples undergo fewer actual cycles of PCR before reaching saturation relative to the end-point experimental samples lacking the contamination, resulting in an exaggeration of the PCR bias between samples. While excess contaminating plasmid causes a bias, elimination of this contamination abolishes the relationship between GC content and Log_2_FC (Figure 4D). The abundance of contaminating plasmid in our mock screen was lower than we had seen in previous experiments, and, correspondingly, we observed only a modest, though statistically significant, correlation between GC content and Log_2_FC. Nevertheless, we were able to abolish that correlation by normalizing our screen sample to reference conditions that did not exhibit contamination, and thus contained the same number of copies of template as the screen sample. Accordingly, using excess copies of plasmid DNA to normalize screen samples resulted in pronounced bias, while no bias was observed when using linearized plasmid diluted into gDNA such that template copy number was equivalent to screen samples. These observations confirm that variations in template abundance between experimental and normalization samples cause artifactual GC bias, and indicate that, unless plasmid input is diluted such that the number copies of the template present is equivalent to the number in the transduced cell population, bias against GC-rich templates will be observed. To our knowledge, considerations of the impact on PCR biases of varying template abundance in different time points or samples from screens have not previously been explored.

We also discovered that recombination can occur between unique library elements in retroviral libraries, resulting in uncoupling and reshuffling of BC-ORF pairs. The rate of recombination depends on the length of the homologous region between unique elements. We observed a recombination rate of nearly 30% in our 5′ BC library in which the ORF and BC are separated by 720 bp, while the rate was reduced to ∼ 6% in our 3′BC library where a 96-bp homology region separates the ORF and BC. These observations are consistent with a large body of work describing recombination in wild-type HIV strains during the RT stage of the viral life cycle (Hu *et al.* 2003). Recombination in retroviral vectors resulting in deletion of one or more elements from a single virus construct has been demonstrated previously (ter Brake *et al.* 2008; Mcintyre *et al.* 2009). However, while retroviral recombination has been studied extensively in the field of virology, because genetic screening using multiple genetic elements in a library format is a relatively recent development, intermolecular retroviral recombination resulting in shuffling of elements in a polyclonal population of viral vectors has not yet been reported.

While a 6% recombination frequency is not negligible, this small proportion of the total number of BCs is unlikely to be problematic, as 94% of the signal still accurately reflects the behavior of the proper ORF. In theory, a recombination event which pairs a phenotypically potent ORF, such as *CCND1*, with a neutral ORF, such as a housekeeping gene, may dampen the enrichment signal, but not reverse it. In the rare cases where a BC recombines to an ORF that confers a very strong and opposing phenotype relative to the ORF it was originally paired with, the effect is still likely to be masked by the combined effects of the additional BCs for that ORF. Thus, the ability to measure internal, independent events and assess their reproducibility within the experiment provides additional confidence in the measurements made with this library.

Retroviral recombination has important implications for experimental design when using constructs with more than one unique element. For example, combinatorial RNAi (co-RNAi) is being explored as both a tool for gene discovery and a therapeutic opportunity for combating resistance-prone diseases like viral infections and cancer (Grimm and Kay 2007; Cheng *et al.* 2009; Diehl *et al.* 2014). Efforts at co-RNAi optimization have illuminated one downstream effect of lentiviral recombination tendency, in the observation that one or more elements can be deleted through intramolecular recombination when a promoter sequence is present multiple times throughout a construct, driving the expression of several distinct elements (ter Brake *et al.* 2008; Mcintyre *et al.* 2009). Thus, efforts have moved toward design of constructs containing multiple promoter/shRNA cassettes that provide efficient and consistent expression (Sano *et al.* 2008; Lambeth *et al.* 2010; Mcintyre *et al.* 2011). As pooled co-RNAi screening is emerging as a tool for discovery of genetic relationships, the effects of intermolecular recombination will present challenges for library design. One study has shown that the orientation of the second promoter driving hairpin expression does not impact knockdown efficiency (Lambeth *et al.* 2010). This could enable library designs in which two unique shRNAs or guide RNAs are positioned nearly adjacent to one another, with flanking promoter sequences driving expression of each in opposite directions. Such a design would overcome the challenges of recombination in dual expression systems. However, the extensive perfect hairpins present in shRNAs could act to enhance reverse transcriptase stalling and template switching *in vivo*. Thus, the structure of the elements may play a role in addition to spacing, and such effects should be examined. We have described several sources of error that can contribute to biased results of genetic screens performed in cultured mammalian cells. In addition, we have suggested strategies to mitigate these errors, and demonstrated that the application of these strategies to screens performed in our laboratory substantially diminished bias, and will therefore be of use to the screening community.

## Supplementary Material

Supplemental Material
