# Supplementary material for: Sources of Error in Mammalian Genetic Screens
Source: G3 (Bethesda). 2016 Jul 6;6(9):2781–90. doi: 10.1534/g3.116.030973 (PMC5015935; doi:10.1534/g3.116.030973)
Supplement: Supplemental Material [file supp_6_9_2781__index.html]

Sources of Error in Mammalian Genetic Screens — Supplemental Material 

# Sources of Error in Mammalian Genetic Screens

## Supplemental Material for Sack, *et al*, 2016

**Files in this Data Supplement:**

- Table S1 - 3' BC Screen Normalized Illumina Read Counts. (.xlsx, 5,954 KB)
- Table S2 - TRE BC Screen Normalized Illumina Read Counts. (.xlsx, 5,879 KB)
- Table S3 - Log2FC and GC content of BCs from 5' BC library screen in Fig. 4A. (.xlsx, 5,554 KB)
- Table S4 - Log2FC and GC content of non-targeting shRNAs from Fig. 4B. (.xlsx, 45 KB)
- Table S5 - Log2FC and GC content of non-targeting shRNAs from mock screen depicted in Fig 4D. (.xlsx, 105 KB)
- File S1 - Lentivirus Benzonase Protocol. (.docx, 20 KB)
