# Supplementary material for: Sources of Error in Mammalian Genetic Screens
Source: G3 (Bethesda). 2016 Jul 6;6(9):2781–90. doi: 10.1534/g3.116.030973 (PMC5015935; doi:10.1534/g3.116.030973)
Supplement: Supplemental Material [file supp_g3.116.030973_FileS1.docx]

**Production of Library Lentivirus with Benzonase Treatment**

**Day 1**

1. Seed 293T cells to be 70%-80% confluent in 24 hours. We seed 9x10^6^-1x10^7^ cells per 150 mm tissue culture dish but numbers may vary based on counting method. Scale according to dish area for smaller culture dishes.
   1. Cells are maintained in DMEM (Thermo Fisher Catalog # 11965-092) supplemented with 10% Fetal Bovine Serum and Penicillin-Streptomycin

**Day 2**

**Table 1.** Transfection components by tissue culture dish size.

| **Plate Size** | **Target (Library) Plasmid** | **Packaging Plasmid (Pre-mixed)** | **TransIT-293** | **OptiMEM** |
| --- | --- | --- | --- | --- |
| 150 mm | 8 ug | 8 ug | 48 ul | To 3 ml |
| 100 mm | 3 ug | 3 ug | 18 ul | To 1 ml |
| 6-well | 500 ng | 500 ng | 3 ul | To 200 ul |

Packaging Plasmid: Prepare a mixture of preferred packaging plasmids, to use at 1:1 ratio with target plasmid. We use Gag/pol, VSVg, Rev, TAT, use at 1:1:1:1 ratio.

Note: DNA amounts can be reduced by half with only a 10-20% reduction in viral titer

1. Bring serum free medium (OptiMEM, Thermo Fisher Catalog # 31985070) and TransIT-293 transfection reagent (Mirus Bio Catalog # MIR 2700) to room temperature
   1. It is not necessary to change the culture medium on the 239Ts despite presence of serum and antibiotics
2. Consult Table 1 to determine correct volume of OptiMEM, DNA, and TransIT-293 to use based on cell culture dish size.
3. Prepare complexes, according to manufacturer’s instructions, summarized as follows:
   1. In a sterile tube, add the appropriate amount of OptiMEM
   2. Add the DNA (library plasmid DNA and packaging plasmid DNA mixture), gently vortex to mix
      1. Note: for best results, maxi-prepped plasmid DNA is recommended
   3. Vortex the TransIT-293 and add the appropriate volume (3X total mass of DNA) to diluted DNA mixture
   4. Vortex to mix
   5. Incubate 20-30 min at room temperature
4. Add transfection complexes dropwise to cells. Pipette gently as agitation can dislodge cells from culture dish
5. Incubate cells at 37⁰C for 14-18 hours

**Day 3**

1. Warm 293T culture medium to 37⁰C.
2. Gently remove transfection medium from cells
3. **Very slowly** add fresh culture medium to cells. Use 15-25 ml medium for a 150 mm culture dish.
   1. Note: cells will become dislodged very easily at this stage, so use extreme caution
   2. Note: if working with several plates of cells, process 1-2 at a time
4. Incubate cells at 37⁰C for 24-36 hours.

**Day 4**

1. Transfer culture medium into an appropriately sized, sterile tube
   1. If desired, add fresh culture medium to cells (very gently) and incubate cells at 37⁰C for 6-24 hours. Several additional harvests can be performed in this fashion, but the titer of the recovered fraction will diminish each time.
2. If dead cells/debris are visible in viral supernatant, centrifuge tube at 1000 rpm for 5 min. Remove the supernatant and transfer to a fresh sterile tube, and discard the pellet.
3. Optional: Concentrate the virus and/or filter the virus using 0.45 um filter units.
4. Treat viral supernatant with Benzonase (adapted from Sastry *et al*. 2004)
   1. Add 10X Benzonase Buffer (500 mM Tris-HCl pH 8.0, 10 mM MgCl_2_, 1 mg/ml bovine serum albumin (BSA)) to viral supernatant to a final concentration of 1X
   2. Add Benzonase to final concentration of 100-500 units/ml
      1. Note: optimal concentration of Benzonase will depend on the amount of plasmid used. This can be optimized by further testing
   3. Incubate (with agitation/rotation if possible) at 37⁰C for 30 min. The half life of virus at 37⁰C is approximately 6 hours so extended incubation at 37⁰C will be detrimental to viral titer.*
5. Aliquot virus and store at -80⁰C for future use.

*Note. The efficiency of Benzonase treatment can be determined by qPCR using probes that recognize the backbone of the plasmid vector containing the virus.
